# Oral Health in Palliative Care: An Exploratory Study of Public Dental Practitioners’ Perceptions in Sydney, Australia

**DOI:** 10.3390/healthcare13182380

**Published:** 2025-09-22

**Authors:** Ajesh George, Ariana Kong, Agnivo Sengupta, Amy R. Villarosa, Tiffany Patterson Norrie, Meera Agar, Janeane Harlum, Deborah Parker, Jennifer Wiltshire, Ravi Srinivas

**Affiliations:** 1 Australian Centre for Integration of Oral Health (ACIOH), School of Nursing and Midwifery, Western Sydney University, Penrith, NSW 2750, Australia; ariana.kong@westernsydney.edu.au (A.K.); a.sengupta@westernsydney.edu.au (A.S.); amy.villarosa@anu.edu.au (A.R.V.); tiffany.patterson@westernsydney.edu.au (T.P.N.); ravi.srinivas@health.nsw.gov.au (R.S.); 2 Ingham Institute for Applied Medical Research, 1 Campbell St, Liverpool, NSW 2170, Australia; 3 School of Dentistry, Faculty of Medicine and Health, University of Sydney, Camperdown, NSW 2050, Australia; 4 Translational Health Research Institute, Campbelltown, NSW 2560, Australia; 5 National Centre for Epidemiology and Population Health, ANU College of Law, Governance and Policy, Australian National University, Canberra, ACT 2600, Australia; 6 School of Health Sciences, Western Sydney University, Locked Bag 1797, Penrith, NSW 2751, Australia; 7 IMPACCT (Improving Palliative, Aged and Chronic Care Through Clinical Research and Translation), Faculty of Health, University of Technology Sydney, Ultimo, NSW 2007, Australia; meera.agar@uts.edu.au (M.A.); deborah.parker@uts.edu.au (D.P.); 8 Department of Palliative Care, South Western Sydney Local Health District, Liverpool, NSW 2170, Australia; janeane.harlum@health.nsw.gov.au (J.H.); jennifer.wiltshire@health.nsw.gov.au (J.W.); 9 District Palliative Care Service, South Western Sydney Local Health District, Liverpool, NSW 2170, Australia; 10 Oral Health Services, South Western Sydney Local Health District, Liverpool, NSW 2170, Australia

**Keywords:** palliative, hospice, dental, oral health

## Abstract

**Introduction:** Oral health problems are prevalent among people receiving palliative care, affecting their quality of life. However, little is known about dental practitioners’ perspectives in this setting. Thus, this study aimed to explore the perceptions of public dental practitioners regarding the provision of dental care for people who receive palliative care. **Methods:** An exploratory focus group with 21 public dental practitioners, with a mean of 8.24 years of experience, was conducted in a public oral health service in Sydney, Australia. The focus group was transcribed and analysed using thematic analysis. **Results:** Three themes were identified: (1) enhancing quality of life in palliative care through improved oral health; (2) navigating the systemic and practical challenges of palliative dental care; (3) competent, collaborative, and optimised: a palliative oral care model. Participants highlighted the importance of oral health to quality of life while receiving palliative care. Nevertheless, there were several systematic and practical challenges to delivering appropriate dental care, which included competing priorities among clients, disconnects and gaps in care coordination with palliative care providers, limited training, and adapting treatment planning during appointments. Participants highlighted the need for a new model of care in the future that improved dental practitioners’ competence through comprehensive palliative dental training, included protocols to facilitate interdisciplinary collaboration, and optimised dental treatment planning and appointment scheduling. **Conclusions:** Public dental practitioners in this study demonstrated positive attitudes, but systemic barriers and limited training restrict their care provision. A palliative oral health care model for this setting should include palliative dental training, foster interdisciplinary collaboration, and optimise dental treatment planning.

## 1. Introduction

A primary goal of palliative therapeutic interventions globally is to improve the quality of life of individuals by improving comfort and addressing symptoms at end-of-life [1]. While poor oral health can have a significant impact on the quality of life of people with a life-limiting illness, it is often not prioritised [2,3]. Due to a decline in health, people receiving palliative care may experience xerostomia (dryness of mouth), which can also cause dry lips, changes in taste and speech, increased sensitivity [4], and increased frequency of painful oral infections such as mucositis and candidiasis [5]. Among older people receiving palliative care, one Japanese study found a high incidence of soft tissue oral health problems and poor adherence to the use of removable dentures despite needing them [6]. Good oral interventions for people receiving palliative care include regular oral hygiene; one recent meta-analysis found that toothbrushing combined with chlorhexidine helped to reduce the risk of ventilator-associated pneumonia for palliative patients on mechanical ventilation [7]. Oral health problems may be further exacerbated by the person’s cognitive or physical decline, leading to inadequate oral self-care, which is important to significantly prevent the worsening of oral hygiene and maintain moisture [2,8]. Another Japanese study found that older people with advanced disease progression may also experience tongue inflammation and dysphagia [9], reducing their ability to speak and consume food and drinks. In the long term, this can contribute to malnutrition and weight loss, deteriorating their overall health and quality of life [9].

Dental treatment has the potential to improve the oral health of people receiving palliative care [5]. Some studies have demonstrated that prophylactic professional oral health care, including scaling, professional cleaning of teeth, and brushing instructions, can prevent deterioration in oral health among people receiving palliative care [10]. However, a UK study identified that one of the major barriers individuals experience in receiving professional care is access to dental services [11]. The reasons for limited access to dental care are complex, and among older people in the United States, this may be attributed to lower prioritisation by families, transport difficulties, and increased psychological issues [12].

Despite the need for dental treatment among individuals receiving palliative care, there are some barriers to dental practitioners delivering care. For instance, American dental practitioners have reported that their professional training did not adequately equip them to address the complex needs of people with a life-limiting illness [13,14]. In the US, inadequate training and experience in managing oral health needs during palliative care could contribute to poor access to dental care, especially if few dental practitioners are confident in providing dental treatment [15]. Although the need for dental practitioners to provide palliative dental care is not a new concept, it is becoming increasingly relevant globally in the context of an ageing population where people have greater life expectancies but also more complex chronic medical conditions that influence their oral health [16].

In Western countries, such as the United States and Australia, estimates indicate that 20% of the population will be over the age of 65 by the year 2030 [17]. To meet the evolving health care landscape in Australia, it is important to ensure that dental services are adapted to address the dental needs of people receiving palliative care. However, little is known of the barriers and challenges that dental practitioners face in the palliative care setting in Australia, where they largely work in either public or private siloed clinics with limited integration with primary care services [18]. Although some surveys have identified the practices, needs, and barriers of dental practitioners within a palliative setting in other developed countries [13,14,19], there is currently no published qualitative literature exploring the perceptions of dental practitioners in Australia.

Understanding the experiences and challenges that contribute to the practices and attitudes of Australian dental practitioners to treat people receiving palliative care is an important step to integrating dental care as part of the holistic care in developing a new model of palliative oral health care. Although most dental practitioners work in private settings, advanced disease progression in palliative care often leads to hospital admissions at the end-of-life, which warrants public dental practitioners to provide care. Thus, this study aimed to explore the perceptions of public dental practitioners on providing dental care for people who receive palliative care.

## 2. Materials and Methods

The Standards for Reporting Qualitative Research (SRQR) checklist and reporting guideline were used to guide the reporting of this study [20].

### 2.1. Study Design

A focus group was conducted as part of an exploratory, qualitative descriptive design [21] to explore the perceptions of dental practitioners providing care in the palliative setting, with the purpose of informing a palliative oral health model to implement within the health service setting.

Consistent with the qualitative descriptive design, a pragmatist approach was chosen to align the study’s focus on the participants’ experiences with their dental workplace [21]. This study forms part of a larger project involving the development and implementation of an oral health referral pathway for palliative care settings. The interpretation of the findings of this study is compared with the perspectives of palliative care nurses [22] and medical practitioners [23] published elsewhere.

### 2.2. Context and Population

The focus group was conducted in a private room at a metropolitan hospital in south-western Sydney, consisting of dental practitioners who were practising within the local health district.

### 2.3. Sampling Strategy

A purposive sampling technique was employed as the study aimed to recruit dental practitioners who were working within the public sector. Invitations to participate in the focus group were e-mailed to dental practitioners working within the district in the form of flyers and participant information sheets. Dental practitioners who were interested could choose to attend the focus group, which was scheduled during an in-service session, so that it was at a time designated by the champion at the oral health service.

### 2.4. Consent Statement

Both written and verbal information about the study were provided to all participants before they were invited to participate in an in-person focus group. In the information sheet, participants were informed that non-participation would not impact their working relationship with the health service. All participants had the opportunity to clarify aspects of the study or ask questions prior to providing written, informed consent.

### 2.5. Ethical Considerations

The investigations were conducted in accordance with the principles outlined in the Declaration of Helsinki (1975, revised in 2013). The South Western Sydney Local Health District Human Research Ethics Committee provided ethical approval for the study (HE17/007). The facilitators were not the participants’ supervisor or manager, and neither were they in a position of power relative to the participants. All data and privacy of participants were handled and stored in accordance with the national statement on ethical conduct in human research.

### 2.6. Data Collection

All dental practitioners who expressed an interest in participating in the study joined one focus group (60 min), which was conducted face-to-face in a private room at the hospital. The study was guided by a focus group schedule, which was informed by prior research [5,13,22] and was reviewed by the working group (Supplementary Material S1). The schedule was reviewed by the study team for relevance and clarity and was compared to the focus group schedules used for the nurses and medical practitioners. While none of the public dental practitioners in the health service were involved in piloting the schedule, two members of the working group were trained dental practitioners (A.G., R.S.) with extensive experience in public dental care. One of the members has had over 10 years of experience in clinical management of public oral health services.

The focus group was conducted at a time appropriate in July 2017 for all participants and was audio-recorded. All eligible participants joined the focus group (100% response rate) as it was scheduled during an in-service session. Although the data was collected in 2017, there are currently no qualitative palliative care studies to date that have published the perspectives of dental practitioners in Australia. The exploratory findings of the perspectives of medical practitioners and nurses were published first [22,23] to explore and highlight the challenges related to palliative dental care, and the interpretation of the findings is reported in the present study.

It was facilitated by the lead author (A.G.), who is a public oral health expert and a trained dental practitioner, and another co-author (A.R.V.), who is a health promotion researcher. Prior to the focus group, relevant demographic information was obtained from all participants. Besides the participants and researchers, no other persons were present for the focus group.

As all of the dental practitioners at the oral health service were present at the focus group, no further focus groups were conducted. Although a single focus group is a limitation of this study, the discussion was rich as the participants’ demographics were homogeneous (e.g., majority female, all public dental practitioners) and they all had an existing working relationship with one another, facilitating the focus group discussion. As the dental practitioners had varied levels of experience, reflected by their title as a senior or regular dentist, this enriched the discussion. As exploratory studies are typically used to explore insights in under-researched areas, reaching data saturation was not a goal of the data collection.

### 2.7. Data Analysis

The audio recording from the focus group was professionally transcribed and de-identified prior to analysis. The transcript was imported into NVivo 12 (QSR International, Melbourne, Australia), a program designed to assist in analysing qualitative research by manual coding and sorting of text. An inductive approach was utilised to undertake a thematic analysis of the focus group to develop sub-themes and themes. As part of the first phase of the six-phase framework outlined by Braun and Clarke [24], the authors familiarised themselves with the data by listening to the recordings and reading the transcript. For the second phase, two authors (A.R.V., A.K.) independently coded the transcript, using a data-driven approach to coding. In phase three, these codes were sorted and combined by A.R.V., A.K., and A.G. to develop three preliminary themes and nine sub-themes along with extracted codes in relation to these themes. For phase four, the candidate themes were reviewed by all authors and refined through a consensus meeting. These themes were further defined and re-named in phase five by A.R.V., A.K., and A.G. For phase six, the write-up of the report was led by A.R.V., A.G., and A.K.

### 2.8. Rigour

Peer-coding and debriefing were used to improve the rigour and trustworthiness of the analysis [25]. To develop the coding structure, two researchers (A.R.V., A.K.) independently developed the coding structure and discussed the constructed themes with a third researcher (A.G.). The process of generating the coding structure and theme development contributed to dependability. Integrating peer debriefing, by engaging a third colleague to review interpretations, informed the findings and strengthened the confirmability of the findings. The themes were also discussed with the larger team to increase the reliability of the interpreted data.

## 3. Results

### 3.1. Demographics

A total of 21 dental practitioners participated in the focus group (Supplementary Material S2). Among these participants, 17 identified as female. Although the majority were dental practitioners (*n* = 18), a few were also senior dental practitioners (*n* = 3). The mean age of the sample was 41.57 years (SD 9.06), with the mean years of experience being 8.24 years (SD 6.07). All dental practitioners had at least a bachelor’s degree; however, four participants also had a graduate certificate (*n* = 1), master’s (*n* = 2), or other (*n* = 1). Three broad themes and nine subthemes were generated following thematic analysis (Table 1).

### 3.2. Theme 1: Enhancing Quality of Life in Palliative Care Through Improved Oral Health

Dental practitioners emphasised the importance of oral health in enhancing the quality of life in palliative care. While poor oral health was prevalent among people in palliative care, dental practitioners discussed that improving the quality of life required autonomous decision-making of patients regarding their oral health. Good oral health and quality of life were also connected to the enjoyment of food, which supports adequate nutritional intake and ultimately helps prevent rapid decline.

#### 3.2.1. Promoting Autonomous Decision-Making to Improve Quality of Life

Participants were aware of the prevalence of poor oral health among palliative care patients. They shared some of their experiences treating patients both in the clinic and in the patients’ homes. Dental treatment was based on shared decision-making with patients to respect their autonomy to provide care that patients felt improved their quality of life. Two participants, for example, offered contrasting patient perspectives on whether dentures would improve their quality of life at end-of-life:

“*I had a patient in the clinic who had cancer… Doesn’t want to have any extractions…So, I did a lot of fillings for her…She didn’t want denture[s]. She doesn’t want to go through all that.*”

(D2)

“*I was asked to see a patient in their home who was dying of cancer…They had about two months to live, but they felt their quality of life was going to be improved by having a denture for that last two months of their life.*”

(D15)

Dental practitioners reflected on the importance of patient autonomy and control over their oral health, which they believed gave patients some dignity at end-of-life.

“*So, they feel that even though they’re going through this process of the end stages of their life, they feel that they have a bit of dignity—a bit of control over that situation.*”

(D1)

#### 3.2.2. Connecting Oral Health to Enjoyment, Nutrition, and Preventing Decline

To improve quality of life, several dental practitioners highlighted the importance of oral health in enabling patients to enjoy food during the final months of terminal illness. Participants noted that many prescribed medications impaired cognitive function and caused dry mouth, compounding oral health issues, especially for patients undergoing radiation therapy.

One practitioner explained the following:

“*A lot of medications keep them a little bit depressed as well. So, yeah, they forget to take care of oral health—brushing basically. The medicines involved can cause dry mouth.*”

(D5)

The dental practitioners connected oral health to enjoying meals was seen not only as a source of satisfaction but also as a meaningful aspect of comfort at the end-of-life. Maintaining food intake was also linked to receiving adequate nutritional intake for patients, which was seen as critical to preventing rapid decline in this population. One dental practitioner provided both perspectives:

“*It [good oral health] improves your quality of life in those terminally ill last months… It gives you satisfaction all the way to the end, at least you can eat something good if you’re going to die [unclear]*”

(D3)

“*If they don’t get good nutrition or somethings stopping them from having this proper medication, nutrition’s going to go downhill further and sooner.*”

(D3)

### 3.3. Theme 2: Navigating the Systemic and Practical Challenges of Palliative Dental Care

Dental practitioners experienced several challenges in providing care to people receiving palliative care.

#### 3.3.1. Advocating for Oral Health as a Priority in Palliative Care

Some dental practitioners emphasised the importance of oral health, recognising that poor oral hygiene could exacerbate existing health conditions. However, participants also acknowledged the challenge of advocating for oral care when patients were more focused on their primary medical condition and general health. Some shared similar views and observed that while oral health was a low priority for many patients, many patients did not recognise the impact of their oral health on their overall medical condition:

“*They keep low priority for the dental issue, because their main thing is their medical condition…*”

(D8)

“*The challenge of keep—like make them keep good oral hygiene when they are worry[ing] about their life, about the cancer they have—and then they don’t think about their oral hygiene…that also will affect on their health or they’re going to have gum disease or whatever. That would increase their problems.*”

(D7)

#### 3.3.2. Adapting Dental Care to Unpredictability, Accessibility, and Fatigue

Due to the complex nature of their medical condition, dental practitioners identified that there were additional challenges. Since patients were medically compromised, one dental practitioner noted the need to deviate from the usual clinical dental practice guidelines and tailor them to palliative care. Nevertheless, the nature of palliative care made it difficult for dental practitioners to plan treatment timelines. One dental practitioner observed the challenge of developing dental management plans due to unpredictable prognoses, whereas another dental practitioner noted that the capacity for self-care among many patients receiving palliative care would be limited.

“*[we] have to deviate from the norm, as in medication and things…the problem is the long-term planning as well. Because you don’t know how long they’re going to last. It might be a week, it might be two months maybe.*”

(D9)

“*they don’t have the strength to take care of themselves.*”

(D4)

Physical accessibility to dental services was another layer of challenge in providing dental treatment. The participants also identified different issues with accessibility. Limited accessibility was largely related to the availability of community transport; however, even when community transport services were available, patients also had physical restrictions in the dental chair.

“*Because sometimes…community transport cannot bring them to the clinic on time. Or they themselves can’t access it or, do you know what I mean.*”

(D1)

“*You might not be able to recline them to position that you want to if you’ve got them in the dental chair, because of certain illnesses that they have.*”

(D4)

Patients’ physical limitations often resulted in fatigue, which affected the duration and frequency of dental appointments. While shorter dental appointments were necessary to manage their energy levels, this sometimes led to the need for more frequent dental visits. Some practitioners discussed the need to reduce extended treatment sessions while minimising the burden of frequent appointments. Another practitioner also highlighted the importance of providing emotional support or counselling during dental treatment, and the time taken to ensure holistic patient care.

“*…in one hour, they will get tired. They can’t handle more than 60 min.*”

(D7)

“*…definitely need more than 40 min. Otherwise, the patient will be coming and going back and forth.*”

(D13)

“*…physical disability or psychological issues or their need to talk sometimes and we need to give them more sympathy and time for the whole treatment.*”

(D13)

#### 3.3.3. Disconnects and Gaps in Care with Other Health Services

Dental practitioners also discussed the disconnect of care between the dental service and other health services. Some shared the difficulties in contacting patients’ specialists and the lack of information from other health professionals on the implications of the patient’s life-limiting condition on their dental condition. In contrast, one practitioner highlighted the role of general practitioners (GPs) to educate patients receiving palliative care about the side effects of medications on their oral health.

“*Trying to chase down a specialist at a hospital regarding a patient is very, very hard.*”

(D1)

“*Sometimes we treat patients with [polio] for example who have no idea that they should have checked their teeth beforehand. I think it’s the GP’s responsibility to educate them about what complications or side effects their medications have on their oral health which is deficiency. We have a big deficiency.*”

(D13)

Other health services seemed to provide people receiving palliative care little support in navigating access to dental services. A dental practitioner shared one instance where a patient had to call over 20 other health services before they were directed to the correct oral health service, inferring that the limited information about oral health services was a difficulty.

“*[they] had to ring around 20 to 30 different places before they were directed to our [oral health] service. So, there is that barrier as well.*”

(D1)

Furthermore, another dental practitioner reflected that access to dental treatment was limited in the public dental setting to patients with a Health Care Card (concession card, or ‘Centrelink card’), irrespective of whether they were receiving palliative care.

“*For those who don’t have [a] Centrelink card, like those who have cancer but unfortunately, they are not on Centrelink, so they won’t be able to have a treatment. I don’t know if we can get any exemption for those people to be treated in public service—[if] they don’t have patient card or health care card.*”

(D7)

#### 3.3.4. Incidental Exposure to Palliative Dental Training

Dental practitioners recognised their lack of structured palliative dental training during their undergraduate curriculum or professional development workshops, citing that their experience and exposure to palliative care “*would only be incidental*”. Consequently, they discussed how their lack of formal palliative dental training affected their confidence in treating this population. Knowledge related to palliative care dental treatment during their undergraduate training was largely theoretical, which made it difficult to translate into real-world clinical dental practice.

“*…I don’t have the experience in oral health and palliative care.*”

(D11)

“*What palliative care is just book-based knowledge for me personally. I don’t have any like real life experience in [comforting] those patients. Which makes it hard. Whatever I say it’s just knowledge from the book—rather than in personal experience*”

(D12)

### 3.4. Theme 3: Competent, Collaborative and Optimised: Palliative Oral Care Model

Participants identified various gaps and potential strategies to inform a palliative oral care model. These strategies include the development of comprehensive training for dental practitioners to improve competency in palliative dental care. There was also the need to implement protocols with the multidisciplinary team to ensure that dental treatment would be streamlined. Finally, services should consider optimising dental treatment and appointment scheduling for people receiving palliative care to ensure appropriate and timely dental care.

#### 3.4.1. Developing Comprehensive Palliative Dental Training

Participants discussed in detail the need for the development of comprehensive training in palliative care for dental practitioners to improve competency. Participants identified various aspects of the palliative dental training that could be included, such as education on the aims of palliative treatment and determining when treating the symptoms, rather than the underlying issue, would be more beneficial.

“*…we need to have some overall training around what it actually means, what the aims are. What is symptomatic treatment versus we might have a sense of something that you feel needs to be done…*”

(D11)

Dental practitioners also emphasised the need for training to provide specific palliative care advice based on patients’ engagement and priority of their oral health, as well as strategies to improve the individual’s motivation. In contrast, one participant also mentioned the need for training to better equip dental practitioners to provide timely and appropriate counselling to patients during dental appointments.

“*…pick up from them [patients] to see whether they are actually vested in their oral health or not…how you can motivate them*”

(D1)

“*We need … more training in psychological things. Because I believe for dental part, we all have reasonable experience. But how we deal with that person emotionally and psychologically, that’s what I think we need to know more.*”

(D7)

Dental practitioners also reported on the need for training to address conflicting priorities between families and patients. One dental practitioner explained that some family members were more concerned about the best clinical outcomes, irrespective of their impact on the patient’s quality of life. Training should stipulate steps to assess and manage oral health in the context of palliative care to ensure that symptoms of discomfort are managed to enhance the quality of life of the patient while counselling and supporting family members.

“*Sometimes their family members are more concerned than the patient. The patients are concerned about living their life. But the family…want them to have perfect teeth and dentures and all that*”

(D10)

“*First we have to assess the oral health, see what the needs are…if they have only a month or two, you’re going to extract all their teeth and give them dentures and the patient doesn’t want it, then you have to calm down the family members and let them go in peace rather than subjecting them to so much of trauma*”

(D10)

#### 3.4.2. Implementing Protocols to Facilitate Interdisciplinary Collaboration

Dental practitioners identified that the lack of interdisciplinary collaboration with other health professionals in the palliative care team meant gaps in continuity of care for people receiving palliative care. One dental practitioner explained that for people who required urgent dental extractions and who were on medications, such as blood coagulants and antibiotics, having specific clinical protocols or pathways in place would provide clarity on treatment planning. Participants agreed on the need for clearer palliative dental protocols to be communicated to GPs, as misunderstandings around medication administration could disrupt treatment planning timelines.

“*It would be helpful to putting a pathway in place where it’s understood that if a patient has identified as having pain, it may require extraction… They know that we need antibiotic to be taken at the clinic. They know that we need an INR [international normalised ratio, a blood test for clotting] within 24 h.*”

(D11)

“*We still have general practitioners who write a script and say to their patients, yeah, just take this on the morning of your [dental] appointment. They arrive, and we say, ‘Sorry, can’t do it. We have to see you take that medication.*”

(D11)

Participants discussed that if referrals were accompanied by a full medical history to facilitate the dental appointment, it would streamline the dental appointment. These comprehensive referrals would also minimise problems around delays in identifying which dental patients receive palliative care, knowing specific precautions, and reducing the time taken to obtain the patient’s medical history.

“*That this patient is being referred to you by me. He is terminally ill. He has got all these medications going through and these are the precautions you might have to take when you are treating this patient. So, we are prepared for that. We don’t need to go through with the patient all that, When you had the cancer? How many radiations? How many chemo? It takes a lot longer to take that medical history. By the end you have nothing you have done for that patient. Just an assessment.*”

(D2)

When asked about how teledentistry could potentially play a role in the current systems in place, one dental practitioner mentioned that teledentistry could increase efficiency in triaging patients, scheduling appointments, and developing treatment plans prior to seeing the patient. It was also mentioned that teledentistry could provide an alternative pathway for patients who were less mobile or had accessibility issues.

“*I think it sounds like a more thorough system of triage… it’s about setting up pathways where there are identified people and it can become part of the roster. Say, for example, there was someone coming in, then when that person is triaged through the intake centre, through our now defined pathway, it would be identified that maybe this person is suitable, can’t travel and therefore you have a time rostered where we can sit in front of a computer, be on the end of a phone and actually give some as you say in real time feedback.*”

(D11)

#### 3.4.3. Optimising Dental Treatment and Appointment Scheduling

Dental practitioners identified the need to optimise and coordinate dental treatment to improve dental outcomes and quality of life while ensuring patient comfort. One participant reflected that within the initial 60 min dental consultation, it was important to provide immediate symptomatic relief and ongoing management advice to family caregivers:

“*But we may be able to achieve quite a lot in a 60 min initial appointment…we don’t know how easy it is for that person or their family members to bring them…even if we are just talking about or teaching the family members about how to provide oral care…you might be able to get something done in terms of the relief of immediate pain and some ongoing advice if you have long enough.*”

(D11)

Dental practitioners were also concerned that patients who were near end-of-life may not be able to receive appropriate treatment and needed expedited or prioritised access to the service. However, the current system scheduling does not allow for additional appointments for clients receiving palliative care. One dental practitioner described an experience where this oversight in the system’s scheduling missed an opportunity to improve the oral health-related quality of life for one of their patients:

“*If you know that we’re going to see some [terminally ill] patients like that on regular basis, we should have some extra spots … available for them so that we can book them quicker. Not like four weeks away. We had one patient like that, and we got a phone call from her daughter saying I just want to cancel his appointment because he’s passed away. You feel like your day is gone. You saw that patient four weeks away and you told them that you were going to build up this tooth. He was very happy that he is going to have this tooth and now he’s gone. Because it was four weeks. Maybe if I did it on the next day, he would have stayed at least three weeks with it.*”

(D2)

## 4. Discussion

Dental practitioners in this study shared positive attitudes towards the role of oral health in improving the quality of life and the need to prioritise dental treatment in the palliative setting. Nevertheless, the findings suggested a tailored palliative model of oral health care in this Australian metropolitan service that would address key challenges that impede the delivery of appropriate and timely dental care. Good oral health in palliative care was already perceived as important; it was a matter of promoting patient autonomous decision-making, dignity, and quality of life. Among those who had experience providing palliative dental treatment, their focus was on patient-centred care and autonomy, and their treatment goals were based on the client’s needs. Such views align with the cornerstone of palliative care management, which is to manage the relief of symptoms, such as pain or discomfort, for patients [26]. According to Mulk, Chintamaneni, Mpv, Gummadapu, and Salvadhi [5], both pain management and early clinical diagnosis are key considerations in the prevention and minimisation of oral health problems among people receiving palliative care. Nevertheless, participants identified several complex challenges, at both the systems level and individual practical level, that limited the delivery of appropriate palliative dental planning and treatment.

From a systems perspective, there was a lack of integration between palliative care services and the oral health services, and no evidence-based protocols were implemented to guide dental treatment planning in this service. Timely and prompt access to appropriate dental care was a key concern among the dental practitioners since the limited physical mobility of many patients was a barrier to delivering dental treatment, which was also found in England [11]. Since palliative care services did not coordinate with oral health services, access to medical records and advice to inform dental treatment planning was restricted, further delaying the provision of treatment to alleviate symptoms and patient concerns. Due to the prevalence of oral health problems among people receiving palliative care, early admission dental referrals and treatment are necessary to ensure timely intervention, which can improve patient comfort and quality of life [27]. In a related study published by the authors [23], palliative medical practitioners also identified the need to refer clients for dental treatment, but reflected on the limited referral pathways from palliative to dental services. Kong, George, Villarosa, Agar, Harlum, Wiltshire, Srinivas, and Parker [22] also explored the perspectives of palliative care nurses and similarly suggested that a trained registered nurse could be designated as an oral health coordinator. Future policies and programs should integrate a coordinator or champion to facilitate the referrals of palliative care clients to dental services, supporting the role of dental practitioners.

Another challenge was that no evidence-based protocol was implemented within the system to direct dental treatment planning and management, despite the complex needs of clients receiving palliative care. Disseminating a protocol could aid dental practitioners in managing client priorities [28], especially since experience in palliative dental care and available staff time [29] among dental practitioners may be limited. The need for palliative oral health care protocols based on existing guidelines was also found in the study of palliative care nurses [22]. A recent UK study found that dental practitioners tended to emphasise the biomedical considerations in treatment decision-making for people living with dementia, and would overlook the psychological or social implications of dental care [30]. Most palliative oral health guidelines have focused on general oral care for primary care providers [31] or carers [32], whereas specific palliative dental treatment guidelines for dental professionals are limited [33]. Although Silva, Bodanezi, Chrun, Lisboa, de Camargo, and Munhoz [2] developed an evidence-based protocol for dental treatment planning among terminal cancer patients, a similar dental treatment protocol for people receiving palliative care more broadly should be developed for clinicians, alongside tailored recommendations for the Australian context.

The call for both formal and practical training to better prepare dental practitioners for palliative dental care management was also discussed, which has also been confirmed by Kong, George, Villarosa, Agar, Harlum, Wiltshire, Srinivas, and Parker [22], and by Villarosa, Agar, Kong, Sousa, Harlum, Parker, Srinivas, Wiltshire, and George [23]. Currently, no undergraduate dental training programs in Australia [34] or elsewhere [13,34] adequately prepare graduates to manage clients receiving palliative care, and no continuing professional development programs in palliative dental care exist to upskill existing Australian dental practitioners. Training programs would need to focus on both the clinical and psychosocial aspects of well-being during palliative care and at end-of-life, which was similarly reported in the United States [13]. Among dental hygienists in Japan, many lacked the confidence and knowledge to manage dental treatment and planning among people with advanced cancer receiving palliative care [35]. Future palliative dental training programs will need to clearly articulate treatment goals for people receiving palliative care, provide guidance on managing family expectations, and increase understanding of the psychological needs of clients. Counselling family and carers is important as they often lack knowledge about oral health and the tools to provide oral hygiene for people receiving palliative care [36]. These areas indicate that to implement changes in practice, future training development would need to be interdisciplinary, involving both palliative primary care providers and social workers.

Developing an interdisciplinary model of palliative oral health care in this setting may need to consider several factors. The model could include comprehensive palliative dental training, appropriate oral health protocols to facilitate interdisciplinary dental referrals and better manage the needs of people receiving palliative care, and strategies to optimise dental treatment and reduce the number of dental appointments. IT support systems that enable the secure sharing of patient information present a potential avenue that could facilitate integrated dental care [37]. In contexts where such systems are not in place, other studies have suggested that a non-dental oral health champion or coordinator, trained by a dental professional, could promote oral health care through education and by facilitating dental referrals [2,22]. Kong, George, Villarosa, Agar, Harlum, Wiltshire, Srinivas, and Parker [22] similarly identified the need for a non-dental champion, such as a nurse. In Australia, integrated oral health models using non-dental professionals have been implemented in other populations with demonstrated effectiveness and could be explored as a potential approach for people receiving palliative care [38,39].

Employing an oral health coordinator could also facilitate teledentistry consultations to mitigate the need to physically access services. In the literature, palliative care nurses identified some limitations with teledentistry, including unreliable internet coverage and the cost of implementation [22]. However, following the social mobility restrictions during the COVID-19 pandemic, receptiveness to telehealth platforms has increased across different population groups [40]. Teledentistry could be implemented as a triage model by dental clinicians to identify and prioritise patients who require the most urgent care more efficiently [41,42]. A recent systematic review identified that teledentistry is an effective tool for dental referrals, treatment planning, and treatment viability [43]. Trained dental clinicians could then provide appropriate oral health recommendations and advice for people who require less urgent care. A potential model that could inform future planning of programs is the Seattle Care Pathway for older patients, which uses a matrix that outlines recommendations for oral health assessment, prevention, treatment, and communication, based on a person’s level of dependency and need [44].

While some findings, such as the gaps and disconnects in care with other health services and the lack of formal dental training for dental practitioners in the undergraduate setting or in professional development workshops, confirm the existing knowledge in this area, these results also strengthen the validity of this focus group discussion. Nevertheless, this study also offers novel insights into the content of future palliative dental training programs and oral health protocols to reduce gaps in palliative care, and also highlights some of the systemic and practical challenges that should be further explored and addressed in future research.

## 5. Limitations

There were a number of limitations to this study. While only one focus group of dental practitioners from the same public oral health service participated, our findings are intended to be compared with the perspectives of palliative nurses and medical practitioners [22,23]. While participants were selected purposively, no additional variation criteria (such as years of experience) were specified. There may have also been a potential bias as the study involved voluntary participation, with a single focus group instead of multiple focus groups. The exploratory nature of this work from a single health district site also limits the generalisability of the findings. Thus, the diverse views of dental practitioners at other sites or countries should be further explored in future research.

While the study was conducted in 2017, potentially limiting its relevance, the findings remain relevant to contemporary contexts. Since the study was published, no other qualitative studies have explored the perspectives of Australian dental practitioners on palliative dental care. The number of palliative care episodes has increased by 37% since 2015–2016 [45], representing an increased need in dental referrals from palliative care providers to dental clinics that remains insufficiently addressed. The advent of COVID-19 has since accelerated the adoption of teledentistry services during the pandemic [40,42], but these services did not include direct referral pathways from palliative care services to dental services. Moreover, teledentistry services were not sustained since the pandemic restrictions were lifted, highlighting the ongoing relevance of these findings to the palliative dental context.

Although there may have been a potential bias of desirability in participants’ responses, dental practitioners were informed that their participation or non-participation in the study would not affect their relationship with their employer or the research team. The facilitators nevertheless ensured that all participants had an opportunity to voice their opinions, and there was a reasonable number of people who participated. There was also a lack of participant validation of themes due to scheduling constraints.

## 6. Conclusions

Little is known about the perspectives and challenges of dental practitioners, particularly in Australia, on dental treatment planning and management for people receiving palliative care. The findings from this exploratory study suggest that while dental practitioners in the public health service have generally positive attitudes towards palliative oral health care, there may be key barriers within the system and in their training that may limit the extent to which they can provide adequate care. To address some of these challenges, future research should explore other sites to confirm or contrast these findings through larger studies to clearly identify what strategies need to be implemented to inform a truly integrated model of care between palliative and oral health services. Further research should also improve interdisciplinary palliative training alongside appropriate protocols to guide dental treatment. Future programs and policies should also consider integrating a coordinator to streamline the referral of clients to dental services and employ the use of teledentistry to triage and prioritise people who may require more urgent dental care.

## Figures and Tables

**Table 1:** Themes and sub-themes from analysis.

Themes	Sub-Themes
Enhancing quality of life in palliative care through improved oral health	Promoting autonomous decision-making to improve quality of life
	Connecting oral health to enjoyment, nutrition, and preventing decline
Navigating the systemic and practical challenges of palliative dental care	Advocating for oral health as a priority in palliative care
	Adapting dental care to unpredictability, accessibility, and fatigue
	Disconnects and gaps in care with other health services
	Incidental exposure to palliative dental training
Competent, Collaborative and Optimised: palliative oral care model	Developing comprehensive palliative dental training
	Implementing protocols to facilitate interdisciplinary collaboration
	Optimising dental treatment and appointment scheduling

## Data Availability

The original contributions presented in this study are included in the article in the form of supporting quotes. Further inquiries can be directed to the corresponding author. Raw data are not publicly available due to ethical restrictions related to privacy and confidentiality.

## Supplementary Materials

The following supporting information can be downloaded at: https://www.mdpi.com/article/10.3390/healthcare13182380/s1, S1: PALLIOH Dental Focus Group Schedule; S2: Individual Participant Demographic Table.

## Abbreviations

The following abbreviations are used in this manuscript:

GP	General Practitioner
SD	Standard deviation
SRQR	Standards for Reporting Qualitative Research

## References

[1] Rietjens J.A.C., Sudore R.L., Connolly M., van Delden J.J., Drickamer M.A., Droger M., van der Heide A., Heyland D.K., Houttekier D., Janssen D.J.A. (2017). Definition and recommendations for advance care planning: An international consensus supported by the European Association for Palliative Care. Lancet Oncol..

[2] Silva A.R.P., Bodanezi A.V., Chrun E.S., Lisboa M.L., de Camargo A.R., Munhoz E.A. (2023). Palliative oral care in terminal cancer patients: Integrated review. World J. Clin. Cases.

[3] Ezenwa M.O., Fischer D.J., Epstein J., Johnson J., Yao Y., Wilkie D.J. (2016). Caregivers’ perspectives on oral health problems of end-of-life cancer patients. Support. Care Cancer.

[4] Gorges J., Wehler B., Krüger M., Singer S. (2018). Oral health-related quality of life in cancer patients. Laryngorhinootologie.

[5] Mulk B.S., Chintamaneni R.L., Mpv P., Gummadapu S., Salvadhi S.S. (2014). Palliative dental care—A boon for debilitating. J. Clin. Diagn. Res..

[6] Furuya J., Suzuki H., Hidaka R., Koshitani N., Motomatsu Y., Kabasawa Y., Tohara H., Sato Y., Minakuchi S., Miyake S. (2022). Factors affecting the oral health of inpatients with advanced cancer in palliative care. Support. Care Cancer.

[7] Sozkes S., Sozkes S. (2023). Use of toothbrushing in conjunction with chlorhexidine for preventing ventilator-associated pneumonia: A random-effect meta-analysis of randomized controlled trials. Int. J. Dent. Hyg..

[8] Okamoto M., Tanaka H., Tamura S., Kanamori D., Yokoi M., Usui M., Yoshida M. (2025). Effects of oral care in terminal cancer patients shortly before death. Gerodontology.

[9] Matsuo K., Watanabe R., Kanamori D., Nakagawa K., Fujii W., Urasaki Y., Murai M., Mori N., Higashiguchi T. (2016). Associations between oral complications and days to death in palliative care patients. Support. Care Cancer.

[10] Saito H., Watanabe Y., Sato K., Ikawa H., Yoshida Y., Katakura A., Takayama S., Sato M. (2014). Effects of professional oral health care on reducing the risk of chemotherapy-induced oral mucositis. Support. Care Cancer.

[11] Delgado M.B., Latour J., Neilens H., Griffiths S. (2021). Oral care experiences of palliative care patients, their relatives, and health care professionals: A qualitative study. J. Hosp. Palliat. Nurs..

[12] Chen X., Chen H., Douglas C., Preisser J.S., Shuman S.K. (2013). Dental treatment intensity in frail older adults in the last year of life. J. Am. Dent. Assoc..

[13] Wilwert M.M., Watkins C.A., Ettinger R.L., Cowen H.J., Qian F. (2011). The involvement of Iowa dentists in hospice care. Spec. Care Dentist..

[14] Sirmons K.L., Dickinson G.E., Burkett T.L. (2010). Teaching end-of-life issues: Survey of U.S. dental schools and dentists. J. Dent. Educ..

[15] Weihermann G.A., Bernhardt F., Brix T.J., Baumeister S.-E., Lenz P. (2024). Role and relevance of dentists in a multiprofessional palliative care team: Results of a cross-sectional survey study. Support. Care Cancer.

[16] Worldwide Palliative Care Alliance, World Health Organization Global Atlas of Palliative Care at the End of Life. https://cdn.who.int/media/docs/default-source/integrated-health-services-.

[17] Roberts A.W., Ogunwole S.U., Blakeslee L., Rabe M.A. The Population 65 Years and Older in the United States. https://www.census.gov/library/publications/2018/acs/acs-38.html.

[18] Sloan A.J., Wise S.L., Hopcraft M. (2024). Primary care dentistry: An Australian perspective. J. Dent..

[19] Venkatasalu M.R., Murang Z.R., Husaini H.A.B.H., Idris D.R., Dhaliwal J.S. (2020). Why oral palliative care takes a backseat? a national focus group study on experiences of palliative doctors, nurses and dentists. Nurs. Open.

[20] O’Brien B.C., Harris I.B., Beckman T.J., Reed D.A., Cook D.A. (2014). Standards for reporting qualitative research: A synthesis of recommendations. Acad. Med..

[21] Doyle L., McCabe C., Keogh B., Brady A., McCann M. (2019). An overview of the qualitative descriptive design within nursing research. J. Res. Nurs..

[22] Kong A.C., George A., Villarosa A.R., Agar M., Harlum J., Wiltshire J., Srinivas R., Parker D. (2020). Perceptions of nurses towards oral health in palliative care: A qualitative study. Collegian.

[23] Villarosa A.R., Agar M., Kong A., Sousa M.S., Harlum J., Parker D., Srinivas R., Wiltshire J., George A. (2024). The perceptions of palliative care medical practitioners towards oral health: A descriptive qualitative study. Palliat. Med..

[24] Braun V., Clarke V. (2006). Using thematic analysis in psychology. Qual. Res. Psychol..

[25] Rose J., Johnson C.W. (2020). Contextualizing reliability and validity in qualitative research: Toward more rigorous and trustworthy qualitative social science in leisure research. J. Leis. Res..

[26] World Health Organization Strengthening of Palliative Care as a Component of Comprehensive Care Throughout the Life Course. https://apps.who.int/gb/ebwha/pdf_files/WHA67/A67_R19-en.pdf?ua=1&ua=1.

[27] Silva M., Santos E.S., Pedroso C.M., Epstein J.B., Santos-Silva A.R., Kowalski L.P. (2024). Prevalence of oral diseases in patients under palliative care: A systematic review and meta-analysis. Support. Care Cancer.

[28] Soileau K., Elster N. (2018). The hospice patient’s right to oral care: Making time for the mouth. J. Palliat. Care.

[29] Herrler A., Valerius L., Barbe A.G., Vennedey V., Stock S. (2022). Providing ambulatory healthcare for people aged 80 and over: Views and perspectives of physicians and dentists from a qualitative survey. PLoS ONE.

[30] Geddis-Regan A., Abley C., Exley C., Wassall R. (2023). Dentists’ approaches to treatment decision-making for people with dementia: A qualitative study. JDR Clin. Transl. Res..

[31] Jones J.A., Chavarri-Guerra Y., Corrêa L.B.C., Dean D.R., Epstein J.B., Fregnani E.R., Lee J., Matsuda Y., Mercadante V., Monsen R.E. (2022). MASCC/ISOO expert opinion on the management of oral problems in patients with advanced cancer. Support. Care Cancer.

[32] Lewis A., Fricker A. Better Oral Health in Residential Care: Professional Portfolio. https://ltctoolkit.rnao.ca/sites/default/files/resources/BOHRC_Professional_Portfolio_Full_Version%5b1%5d.pdf.

[33] Rohr Y., Adams J., Young L. (2010). Oral discomfort in palliative care: Results of an exploratory study of the experiences of terminally ill patients. Int. J. Palliat. Nurs..

[34] Mills J., Kim S.-H., Chan H.Y.L., Ho M.-H., Montayre J., Liu M.F., Lin C.-C. (2021). Palliative care education in the Asia Pacific: Challenges and progress towards palliative care development. Prog. Palliat. Care.

[35] Nakajima N. (2020). Challenges of dental hygienists in a multidisciplinary team approach during palliative care for patients with advanced cancer: A nationwide study. Am. J. Hosp. Palliat. Med..

[36] Prieto D., Palacios M., Wainstein V., Ortuño D., Mora S., Chaparro A., Pascual A. (2025). Knowledge and experiences of oral health care and periodontitis of caregivers of older adults with cognitive impairment. Gerodontology.

[37] Carter K., Chalouhi E., McKenna S., Richardson B. What It Takes to Make Integrated Care Work. https://www.mckinsey.com/~/media/mckinsey/dotcom/client_service/healthcare%20systems%20and%20services/health%20international/issue%2011%20new%20pdfs/hi11_48%20integratedcare_noprint.pdf.

[38] Kong A., Dickson M., Ramjan L., Sousa M.S., Jones N., Srinivas R., Chao J., Goulding J., George A. (2021). Aboriginal health workers promoting oral health among Aboriginal and Torres Strait Islander women during pregnancy: Development and pilot testing of the Grinnin’ Up mums & bubs program. Int. J. Environ. Res. Public Health.

[39] George A., Dahlen H.G., Blinkhorn A., Ajwani S., Bhole S., Ellis S., Yeo A., Elcombe E., Johnson M. (2018). Evaluation of a midwifery initiated oral health-dental service program to improve oral health and birth outcomes for pregnant women: A multi-centre randomised controlled trial. Int. J. Nurs. Stud..

[40] Erikson C., Park Y.H., Felida N., Dill M. (2023). Telehealth use and access to care for underserved populations before and during the COVID-19 pandemic. J. Health Care Poor Underserved.

[41] Ghai S. (2020). Teledentistry during COVID-19 pandemic. Diabetes Metab. Syndr. Clin. Res. Rev..

[42] Maqsood A., Sadiq M.S.K., Mirza D., Ahmed N., Lal A., Alam M.K., Halim M.S.B. (2021). The teledentistry, impact, current trends, and application in dentistry: A global study. BioMed Res. Int..

[43] Gurgel-Juarez N., Torres-Pereira C., Haddad A.E., Sheehy L., Finestone H., Mallet K., Wiseman M., Hour K., Flowers H.L. (2022). Accuracy and effectiveness of teledentistry: A systematic review of systematic reviews. Evid. Based Dent..

[44] Pretty I.A., Ellwood R.P., Lo E.C.M., MacEntee M.I., Müller F., Rooney E., Murray Thomson W., Van der Putten G.-J., Ghezzi E.M., Walls A. (2014). The Seattle care pathway for securing oral health in older patients. Gerodontology.

[45] Australian Institute of Health Welfare Palliative Care Services in Australia. https://www.aihw.gov.au/reports/palliative-care-services/palliative-care-services-in-australia.

