# Supplementary material for: Oral Health in Palliative Care: An Exploratory Study of Public Dental Practitioners’ Perceptions in Sydney, Australia
Source: Healthcare (Basel). 2025 Sep 22;13(18):2380. doi: 10.3390/healthcare13182380 (PMC12469769; doi:10.3390/healthcare13182380)
Supplement: Supplementary file 1 [file healthcare-13-02380-s001.zip › healthcare-3848835-supplementary.pdf]

healthcare

MDPI

Supplementary Materials S1: PALLIOH Dental Focus Group Schedule

- What do you think about the importance of maintaining oral health for palliative care patients - why/why not?
- What are the roles of palliative care clinicians, particularly nurses, in maintaining the oral health of patients in current practice?
- What do you perceive as the facilitators and barriers to implementing the targeted oral health program (risk assessment, prevention, and referral) in the palliative care setting?
- What are your recommendations on overcoming difficulties and barriers to delivering a targeted oral health program in the palliative care setting?
- Do you feel that palliative care clinicians have the knowledge and confidence to provide oral health education, assessment, and referrals to patients?
- What specific further education and training skills are needed for clinicians to provide oral health care programs and referral services?
- What would be the preferred content, duration, and medium of training and education?
- What are clinicians' and dentists' perceptions of using teledentistry in a palliative care setting?
- What do clinicians and dentists perceive as the facilitators and barriers to using teledentistry in the palliative care setting?

Supplementary Materials S2: Individual Participant Demographic Table

Participant	Age	Gender	Position	Years Experience	Highest Qualification
D1	26	Male	Dental Practitioner	3.00	Bachelor
D2	28	Female	Dental Practitioner	1.00	Other - Postgraduate degree
D3	32	Female	Dental Practitioner	3.00	Bachelor
D4	35	Female	Dental Practitioner	1.00	Masters
D5	40	Female	Dental Practitioner	10.00	Bachelor
D6	56	Male	Senior Dental Practitioner	8.00	Bachelor
D7	30	Female	Dental Practitioner	0.67	Bachelor
D8	46	Female	Dental Practitioner	20.00	Bachelor
D9	32	Female	Dental Practitioner	6.00	Bachelor
D10	60	Female	Senior Dental Practitioner	14.00	Bachelor
D11	52	Female	Dental Practitioner	16.00	Bachelor
D12	46	Female	Senior Dental Practitioner	*	Masters
D13	41	Female	Dental Practitioner	9.50	Bachelor
D14	39	Female	Dental Practitioner	7.20	Bachelor
D15	45	Female	Dental Practitioner	10.00	Bachelor
D16	48	Female	Dental Practitioner	6.00	Bachelor
D17	46	Male	Dental Practitioner	14.00	Bachelor
D18	45	Female	Dental Practitioner	12.00	Bachelor
D19	33	Female	Dental Practitioner	1.50	Bachelor
D20	50	Male	Dental Practitioner	20.00	Bachelor
D21	43	Female	Dental Practitioner	2.00	Graduate Certificate

* Missing data.

 https://doi.org/10.3390/healthcare13182380
